# Biomechanics of Spiral Fractures: Investigating Periosteal Effects Using Digital Image Correlation

**DOI:** 10.3390/jimaging11060187

**Published:** 2025-06-07

**Authors:** Ghaidaa A. Khalid, Ali Al-Naji, Javaan Chahl

**Affiliations:** 1 Electrical Engineering Technical College, Middle Technical University, Baghdad 10022, Iraq; ghaidaakhalid@mtu.edu.iq; 2 School of Engineering, University of South Australia, Mawson Lakes, SA 5095, Australia; javaan.chahl@unisa.edu.au; 3 Platforms Division, Defence Science and Technology Group, Edinburgh, SA 5111, Australia

**Keywords:** biomechanics, spiral fracture, immature bone, mechanical testing, periosteum, digital image correlation

## Abstract

Spiral fractures are a frequent clinical manifestation of child abuse, particularly in non-ambulatory infants. Approximately 50% of fractures in children under one year of age are non-accidental, yet differentiating between accidental and abusive injuries remains challenging, as no single fracture type is diagnostic in isolation. The objective of this study is to investigate the biomechanics of spiral fractures in immature long bones and the role of the periosteum in modulating fracture behavior under torsional loading. Methods: Paired metatarsal bone specimens from immature sheep were tested using controlled torsional loading at two angular velocities (90°/s and 180°/s). Specimens were prepared through potting, application of a base coat, and painting of a speckle pattern suitable for high-speed digital image correlation (HS-DIC) analysis. Both periosteum-intact and periosteum-removed groups were included. Results: Spiral fractures were successfully induced in over 85% of specimens. Digital image correlation revealed localized diagonal tensile strain at the fracture initiation site, with opposing compressive zones. Notably, bones with intact periosteum exhibited broader tensile stress regions before and after failure, suggesting a biomechanical role in constraining deformation. Conclusion: This study presents a novel integration of high-speed digital image correlation (DIC) with paired biomechanical testing to evaluate the periosteum’s role in spiral fracture formation—an area that remains underexplored. The findings offer new insight into the strain distribution dynamics in immature long bones and highlight the periosteum’s potential protective contribution under torsional stress.

## 1. Introduction

Soft tissue injuries are the most frequent clinical manifestation of physical abuse in young children, followed by fractures [1,2]. Skeletal fractures are found in up to one-third of children who are the subject of abuse investigations [3]. Children aged 5 years and older accounted for 85% of non-abusive fractures [3]. In contrast, children younger than 18 months accounted for approximately 80% of fractures that were determined to be the result of abuse. According to estimates, non-accidental injuries account for between 25% and 56% of fractures in infants younger than 12 months [3,4]. These high proportions likely result from the limited mobility of infants in this age group and the general expectation that they are protected from physical trauma. Any breach of this protection should be justified by caregivers.

The injury history, fracture location, type and morphology, number of fractures, and the child’s age play significant roles in distinguishing between abusive and accidental fractures [5]. The skull and long bones are the most commonly fractured regions in infants under one year of age, in both unintentional and intentional trauma cases [2,3,6]. Among long bone fractures, humeral fractures are the second most common in infants, following femoral fractures [2]. Non-accidental injuries are estimated to account for 40–80% of long bone fractures in this population [2,4]. Specifically, approximately 43% of humeral fractures and 60% of femoral fractures have been attributed to non-accidental trauma [2,5,7,8]. Femoral fractures resulting from abuse are more common in non-ambulatory infants, whereas falls or unintentional injuries are more often associated with femoral fractures in ambulatory age groups [2].

**Figure 1:**
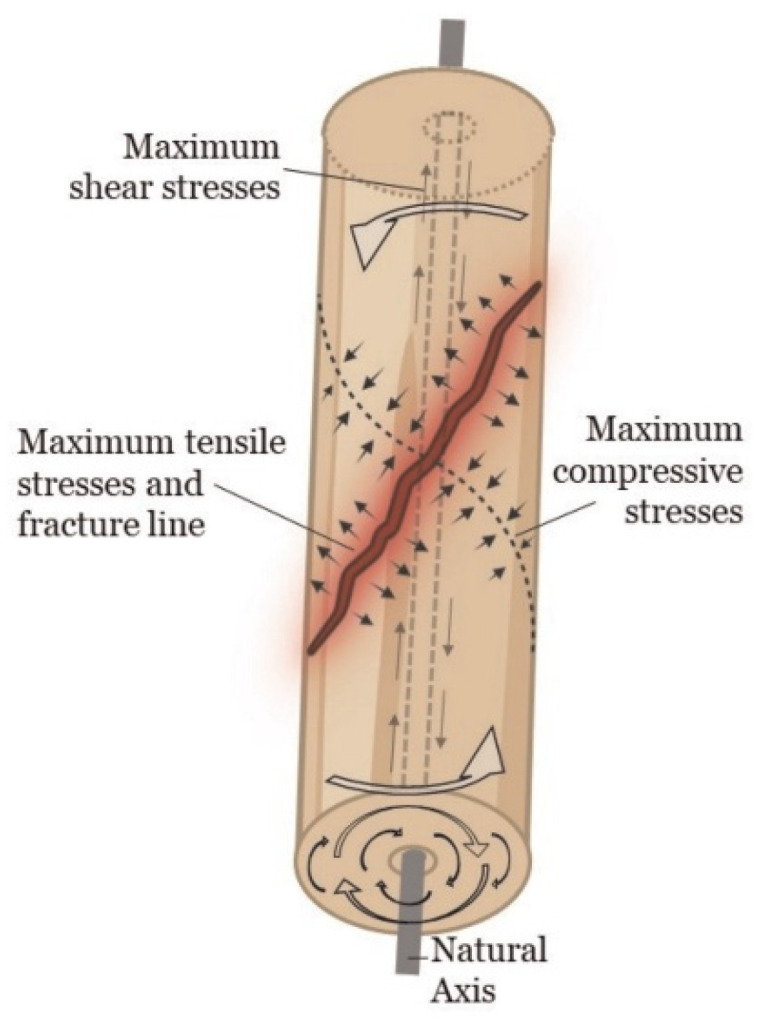
Diagram illustrating the distribution of shear, tensile, and compressive stresses under supraphysiological loading conditions that result in a spiral fracture pattern. Adapted from Alexandre et al., 2024 [11].

A fracture occurs when the internal resistance of a material to deformation is exceeded under applied loading. When a long bone is subjected to pure torsion, it rotates around its longitudinal axis, leading to the formation of a spiral fracture. From a biomechanical perspective, torsional loading generates a combination of shear, tensile, and compressive forces within the bone structure [5,9]. Specifically, torsional forces induce shear stress distributed across the bone’s cross-section. As with bending, the stress magnitude follows a gradient that depends on the distance from the neutral or central axis. Under such loading, the highest shear stresses develop along planes that are both perpendicular and parallel to the central axis. Tensile and compressive stresses align perpendicularly to each other along a diagonal plane relative to the neutral axis, as illustrated in Figure 1. Fracture typically initiates along the plane of maximum shear stress—parallel to the neutral axis—and propagates along the surface of highest tensile stress, producing the classic spiral fracture configuration [2,4,5,6,9,10].

The application of digital image correlation (DIC) in biomechanics has expanded significantly, encompassing the investigation of vascular tissue elongation [12], cartilage behavior [13], and osteonecrosis [14]. Owing to its non-contact nature and high spatial resolution, DIC has become a widely adopted technique for assessing various biomechanical properties across different tissue types.

One of the most common applications of DIC in biomechanics is the assessment of surface stress in bone models under various loading conditions. For example, ref. [15] utilized DIC to measure surface stress on a femur subjected to axial compression, while [16] investigated surface stress on a mouse tibia under similar compressive loading. Furthermore, studies [17,18] applied DIC techniques to analyze composite long bone models exposed to axial compression. Notably, ref. [18] also validated their composite leg models under four-point bending and torsional loading using DIC.

This study employs high-speed digital image correlation (HS-DIC) to investigate the biomechanics of failure in juvenile long bones. Torsional loading is applied to bones from an immature animal model, and the resulting fractures are captured and analyzed using HS-DIC. Throughout the failure process, the technique enables precise tracking of displacement and strain distribution. The study also aims to validate the use of DIC in evaluating surface stress patterns in immature long bones under torsional loading. This approach may contribute to a better biomechanical understanding of spiral fractures in neonatal long bones. While prior research has addressed general fracture mechanics, few studies have examined the specific role of the periosteum in spiral fracture formation using high-speed DIC. This work uniquely integrates advanced imaging and a paired experimental design to elucidate the periosteum’s contribution to torsional fracture behavior in immature bone.

## 2. Materials and Methods

### 2.1. Specimen Preparation

Pearce et al. [19] examined the suitability of various animal species as models for human bone studies. Commonly used species include dogs, sheep, goats, pigs, and rabbits. Only minor differences in bone composition were observed between these animals and humans. Among them, dogs and sheep/goats have demonstrated high similarity to human bone [19]. However, the use of dogs raises ethical concerns due to their role as companion animals. In contrast, sheep present fewer ethical issues and are readily available as a byproduct of the food industry, making them a practical and appropriate choice for this study. Fourth metatarsal bones from skeletally immature female sheep were procured from a local market and used as experimental specimens. Standard procedures for bone preservation were followed, which involved freezing the bones at −20 °C, wrapped in saline-soaked gauze to prevent dehydration [20,21,22].

Bone preparation was carried out by an experienced professor of veterinary surgery with extensive expertise in specimen handling and surgical procedures. The left and right humeri of each pair were preserved intact. In each case, one bone had its periosteum surgically removed, while the contralateral bone retained its periosteum. Assuming anatomical and morphological similarity between paired bones, this design enabled a direct biomechanical comparison between periosteum-intact and periosteum-removed specimens.

To facilitate clarity in sample identification throughout the experiments and figures, each bone specimen was assigned a specific code based on the presence or absence of the periosteum. Specimens labelled with an ‘X’ suffix indicate bones without periosteum, while those labelled with a ‘Y’ suffix indicate bones with periosteum. This coding system was applied consistently across both the primary and secondary test groups. A detailed summary of the sample codes and their group classifications is provided in Table 1. Table 1 summarizes the specimen coding system used throughout the primary and secondary testing phases, categorized by periosteum status. This coding is used consistently across the figures, analysis, and results interpretation.

Calipers were used to measure each bone’s length, mediolateral width, and anteroposterior width. Near the growth plate, a screw was inserted into the distal end of each bone to minimize movement during potting. Wood’s metal—a eutectic alloy with a low melting point (~70 °C)—was used to secure the distal ends of the bones into the center of a 60 mm × 60 mm square steel mold. The alloy was melted using a heat gun to ensure proper fixation.

### 2.2. Digital Image Correlation

This section introduces the terminology and foundational concepts related to digital image correlation (DIC), a non-contact optical method widely used in experimental mechanics. This method relies on the tracking of artificially applied surface patterns to capture deformation under loading.

In the context of digital image correlation (DIC), the term “speckle pattern” refers to an artificially applied, high-contrast, random surface texture used to enable full-field displacement and strain measurements. Although the term “speckle” originates from classical optics, where it refers to interference-based patterns, its usage in DIC is widely accepted in the experimental mechanics literature [12,13]. This clarification is provided to avoid confusion for readers unfamiliar with DIC methodology.

Digital image correlation (DIC) is a non-contact, full-field optical measurement technique used to quantify surface deformation and stress in materials under load. It operates by tracking the movement of distinct points within a high-contrast speckle pattern applied to the specimen’s surface and comparing their positions to a reference (undeformed) image. Using two synchronized cameras in a stereo configuration, DIC enables three-dimensional calculations of strain and displacement [23].

A high-quality speckle pattern is essential for accurate analysis, as the software relies on these features to track surface deformation. Patterns with greater randomness and more distinct features enhance measurement precision [24]. One critical factor affecting accuracy is speckle size; studies have shown that an optimal speckle diameter lies between 2 and 4 pixels, which minimizes displacement bias and maximizes subset fluctuation consistency [25]. Furthermore, speckle density plays a vital role: finer, denser patterns with a higher number of speckles have demonstrated superior performance compared to coarser patterns, as they enable the tracking of more unique features and reduce measurement error [26].

### 2.3. Digital Image Correlation Setup

**Figure 2:**
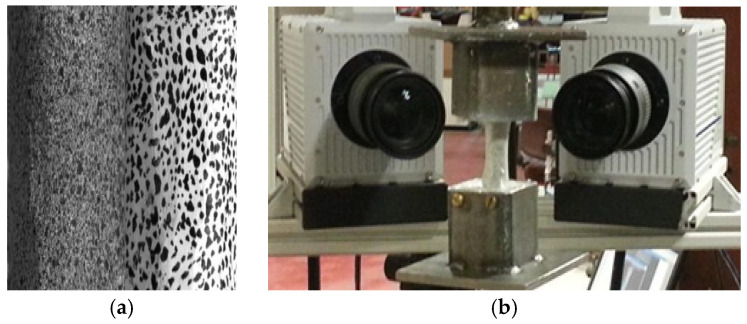
(**a**) Comparison between hand-painted (right) and airbrush-applied (left) speckle patterns on bone surfaces for DIC analysis. (**b**) Experimental setup showing high-speed DIC cameras positioned for capturing full-field strain during torsional loading.

As part of the experimental setup, high-speed DIC cameras (manufactured by Correlated Solutions, Inc., Irmo, SC, USA) were positioned and precisely focused on the speckle pattern applied to the bone surface (see Figure 2). Additional lighting was arranged to enhance exposure and minimize vignetting. A calibration grid with dimensions of 12 × 9 squares (each 5 mm) was used to perform system calibration once the cameras were properly aligned.

Two methods were used to apply the speckle pattern to each potted bone specimen. In the airbrush technique, three sequential layers of cellulose (serving as a matte white primer) were sprayed, allowing each layer to dry completely before the next was applied. Afterwards, a black speckle pattern was airbrushed onto the white base using black acrylic paint. Alternatively, the speckles were applied by hand over the same three white primer layers. The hand-painted method demonstrated better regularity and contrast and was also found to be more consistent and reproducible. Figure 2a illustrates the differences between the two speckle pattern application techniques used in this study.

In the secondary test, bone specimens were repositioned so that their posterior surface directly faced the DIC cameras, allowing for better visualization of the fracture site. To enhance temporal resolution and capture rapid fracture dynamics, the camera frame rate was increased from 1000 to 4000 frames per second. Spiral fractures typically occur within milliseconds, making them difficult to record using conventional imaging systems. Therefore, a high-speed digital image correlation (HS-DIC) setup was employed to capture full-field strain distributions during the fracture process.

This adjustment significantly improved the ability to observe fracture initiation, particularly under higher rotational speeds, and ensured that critical strain patterns were accurately recorded at the moment of failure.

The hand-painted speckle method was employed, and the posterior aspect of the potted bones was oriented toward the DIC cameras. The camera’s frame rate was increased from the default 1000 frames per second to 4000 frames per second to improve temporal resolution.

Deformation and strain data were analyzed using the VIC-3D measurement system (Correlated Solutions Inc., Irmo, SC, USA), which enables full-field, three-dimensional displacement tracking through stereo digital image correlation [23]. Surface strain was calculated as the ratio of the change in length (deformation) to the original length of the bone specimen, following standard strain definitions.

Strain tensors describe the magnitude and direction of surface deformation at specific locations on the specimen. In the VIC-3D system, exx represents normal strain along the *x*-axis, eyy represents normal strain along the *y*-axis, and exy represents shear strain. The system operates using a standard right-hand coordinate system, where the *x*-axis points to the right, the *y*-axis points upward, and the *z*-axis extends outward toward the observer.

The VIC-3D system uses a square-to-parallelogram deformation analogy to define the sign convention for shear strain [23]. When the top edge of the square moves in the positive X direction relative to the bottom edge, the shear strain is considered positive. Conversely, movement in the negative X direction corresponds to negative shear strain.

Torsion occurs when a structure is twisted about its longitudinal axis, typically with one end fixed. Torsional fractures, such as those of the tibia, are commonly observed in sports injuries like football and skiing, where the foot is immobilized while the body continues to rotate. When subjected to torsional loading, a structure develops internal shear stresses, with maximum stress occurring at the outer surface and diminishing to zero at the neutral axis.

The generated torque depends on both the magnitude of the applied force and its perpendicular distance from the axis of rotation. Thus, torque can be mathematically expressed as:

(1) τ=Frsinθ

where τ is the torque (N·mm), F is the applied force (N), r is the lever arm length (mm), and θ is the angle (in degrees) between the force vector and the lever arm.

Each test in this study was conducted using a torsion testing apparatus operating at two rotational speeds: 90°/s and 180°/s. The rotation started at a relative angle of 0°, with testing concluding at 90°. Specimens without periosteum were rotated clockwise to 90°, while specimens with periosteum were rotated counterclockwise to −90°, relative to the same zero reference point.

The potted ends of the specimens were positioned above the lower cup of the hydraulic servo-material testing apparatus. Axial displacement was restricted to ensure that the applied load remained purely torsional, eliminating any compressive or tensile components.

During the initial tests, high-speed DIC cameras were configured to capture the lateral surface of the bones at 1000 frames per second. However, several fractures occurred outside the camera’s field of view. To improve accuracy in the secondary tests, the bones were repositioned with the posterior surface facing the DIC cameras. Additionally, the frame rate was increased to 4000 frames per second to enhance temporal resolution and ensure clear visualization of fracture initiation.

### 2.4. Torsional Loading Protocol

The two angular velocities selected—90 degrees per second and 180 degrees per second—were chosen to evaluate the effect of torsional loading speed on spiral fracture behavior. The selected rotational speeds of 90°/s and 180°/s were chosen to represent two distinct torsional strain rates in an experimental context. While these values do not directly correspond to realistic physiological motions or trauma conditions, they are commonly used in laboratory-based biomechanical testing. This controlled variation enables assessment of rate-dependent behavior in immature bone, particularly for viscoelastic effects under torsional loading.

These values were intended to represent low and high torsional strain rates, respectively, allowing for a comparative analysis of the viscoelastic response and failure properties of immature bone under different loading conditions.

A paired randomization strategy was used to assign bone specimens to each testing condition. Specimens from the same animal were exposed to both low and high strain rate conditions, enabling direct intra-animal comparison and minimizing biological variability.

The primary and secondary tests refer to the two experimental stages conducted in this investigation. In the primary test, bone specimens were subjected to torsional loading using a speckle pattern applied via airbrush and imaged at a frame rate of 1000 frames per second. During this phase, the DIC cameras were positioned to capture the lateral surface of the bones. However, the quality of the data was limited by issues such as fracture events occurring outside the camera’s field of view and insufficient speckle contrast.

To address these limitations, a secondary test was conducted with enhancements in both imaging setup and specimen preparation.

In the primary test, 18 specimens (9 pairs) were tested—half at 90°/s and half at 180°/s. A total of 14 bone specimens were initially prepared for the secondary test. One specimen was excluded prior to testing due to poor speckle pattern quality, leaving thirteen specimens for further study. Of these, two did not develop spiral fractures and were excluded from subsequent analysis. One additional specimen had incomplete torque sensor data, resulting in ten valid specimens being included in the final analysis. In the primary torsional test, the speckle pattern was applied using the airbrush technique. Bones were positioned laterally in front of the DIC cameras, and imaging was performed at 1000 frames per second. For the secondary test, 13 additional specimens were prepared using the same base-speckle method but with two key improvements: (1) specimens were reoriented so that their posterior surface faced the DIC cameras, and (2) the frame rate was increased to 4000 frames per second to enhance temporal resolution and improve the likelihood of capturing fracture initiation. In addition, the speckle pattern in the secondary test was applied manually to improve contrast, consistency, and pattern reproducibility. These refinements collectively enhanced the quality of DIC analysis and enabled more accurate visualization of fracture onset.

The torsion testing instrument recorded multiple parameters, including time, rotation angle, torque, and axial displacement. As illustrated in Figure 2b, the high-speed DIC camera setup was used to capture strain data during torsional loading. From the onset of rotation until bone failure, up to 900 DIC frames were acquired per specimen for analysis. Output data from paired specimens—with and without periosteum—tested at different rotational speeds were analyzed using Student’s paired *t*-test to assess statistical significance. All analyses were conducted using GraphPad 8.4.3software (GraphPad Software Inc., San Diego, CA, USA) with a 95% confidence level. *p*-values were considered statistically significant at ≤0.05.

## 3. Results

### 3.1. Torsional Primary Test

The mechanical performance of 20 bone specimens under torsional loading was evaluated during preliminary investigations by recording and analyzing the mean torque at failure, as presented in Table 2 and Table 3. Two of these specimens did not exhibit spiral fractures and were therefore excluded from the final analysis. Specimens labelled “X” had their periosteum surgically removed and were rotated clockwise around the long axis of the bone. In contrast, specimens labelled “Y” retained the periosteum and were rotated counterclockwise.

### 3.2. Torsional Secondary Test

Similar to the initial investigation, an additional 13 bone specimens were tested during the secondary investigation. Of the fourteen specimens initially prepared, two did not develop spiral fractures and were therefore excluded from the results.

The data collected from the servo-hydraulic testing equipment during the secondary tests are summarized in Table 4 and Table 5. As in the primary test, specimens labelled “X” had their periosteum removed and were rotated clockwise around their longitudinal axis. Specimens labelled “Y” retained the periosteum and were rotated counterclockwise.

**Figure 3:**
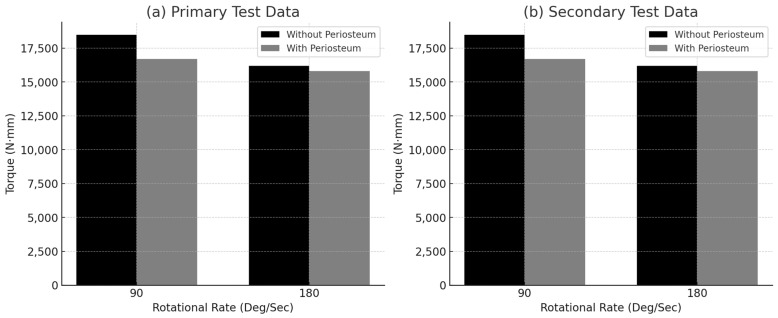
Mean torque at failure for each rotational speed: (**a**) primary test data, and (**b**) secondary test data.

Figure 3 presents the mean torque at failure for both rotational speeds (90°/s and 180°/s) and compares paired bone specimens with and without periosteum. The results indicated that bones with preserved periosteum exhibited lower torque capacity at failure compared to their counterparts without periosteum, suggesting that the periosteum may influence torsional strength by constraining deformation.

The data presented in Table 2, Table 3, Table 4 and Table 5 support this observation, indicating that in 10 out of 14 paired tests, bones with periosteum failed at a lower torque than those without. However, previous research by [27] suggests that the periosteum contributes to bone strengthening, which appears to contrast with the current findings. This discrepancy may be attributed to differences in experimental design, specimen age, or loading conditions.

Both paired and unpaired Student’s *t*-tests were employed to compare fracture torque values between bones evaluated at different rotational speeds. In the primary test at 90°/s (Table 2), the comparison between bones with and without periosteum yielded a *p*-value of 0.7099, indicating no significant difference. In contrast, at 180°/s (Table 3), a statistically significant difference was observed (*p* = 0.0032), with bones lacking periosteum requiring a significantly higher torque to fail.

In the secondary test, the comparison at 90°/s (Table 4) yielded a *p*-value of 0.3, indicating no statistically significant difference. At 180°/s (Table 5), statistical analysis could not be performed due to insufficient sample size.

These findings suggest that rotational speed may influence the detectability of the periosteum’s effect on fracture torque, particularly under higher strain rates where the difference becomes more pronounced.

Although the average torque at failure was higher for bones tested at 180°/s, individual values in Table 2 and Table 3 reveal inconsistencies. The highest torque recorded was 21,088.89 N·mm at 90°/s, which exceeded the maximum value at 180°/s (20,777.28 N·mm). Similarly, one torque measurement at 90°/s (14,592.80 N·mm) was greater than a corresponding value at 180°/s (13,833.83 N·mm). These discrepancies may suggest variability within individual specimens or potential outlier influence affecting the average values.

Unpaired Student’s *t*-tests were performed to assess differences between the datasets. The comparison between Table 2 and Table 3 yielded a *p*-value of 0.4026, while the comparison between Table 4 and Table 5 produced a *p*-value of 0.1627. Both values exceed the 0.05 threshold, indicating no statistically significant differences.

**Figure 4:**
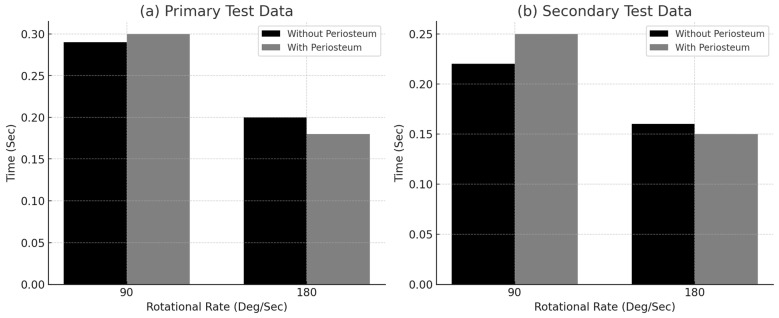
Mean time to failure for each rotational speed: (**a**) primary test data, and (**b**) secondary test.

Figure 4 displays the mean time to failure for specimens tested at both rotational speeds (90°/s and 180°/s), using data from the primary and secondary tests. The comparison also highlights differences between specimens with and without periosteum, showing a tendency for faster failure at higher angular velocities and in bones lacking periosteum.

As illustrated in both graphs, the time to fracture decreased as the rotational speed increased. Specimens tested at 90°/s generally exhibited longer times to failure than those tested at 180°/s, as shown in Table 2, Table 3, Table 4 and Table 5.

Statistical analysis of the time to failure between rotational speeds revealed highly significant results. Comparing Table 2 and Table 3 (primary test) yielded a *p*-value of 0.0002, while the comparison between Table 4 and Table 5 (secondary test) resulted in a *p*-value of 0.0014. Both values are well below the 0.05 threshold, indicating statistically significant differences. These findings align with the viscoelastic properties of bone tissue [20,28], where higher strain rates lead to more rapid failure.

At 90°/s, specimens with periosteum exhibited a longer mean time to failure, as shown in Figure 4. Conversely, at 180°/s, bones without periosteum took longer to fail. However, when comparing specimens with and without periosteum at each rotational speed, no statistically significant differences were found: the *p*-values from Table 2 and Table 4 were approximately 0.9 (*p* > 0.05). Due to limited data in Table 5, statistical comparison could not be performed.

The only statistically significant difference based on periosteum status was found in Table 3 (at 180°/s), where the *p*-value was 0.018, suggesting that bones without periosteum required more time to fail under higher loading conditions.

### 3.3. Digital Image Correlation in Primary Investigation

**Figure 5:**
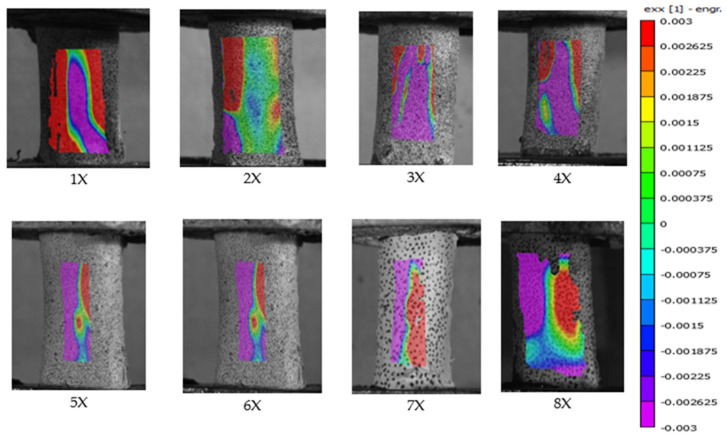
Full-field strain distribution in the X direction (exx) for the “X” specimen obtained via digital image correlation, showing the frame immediately before fracture. The solid blue line indicates the spiral fracture path.

**Figure 6:**
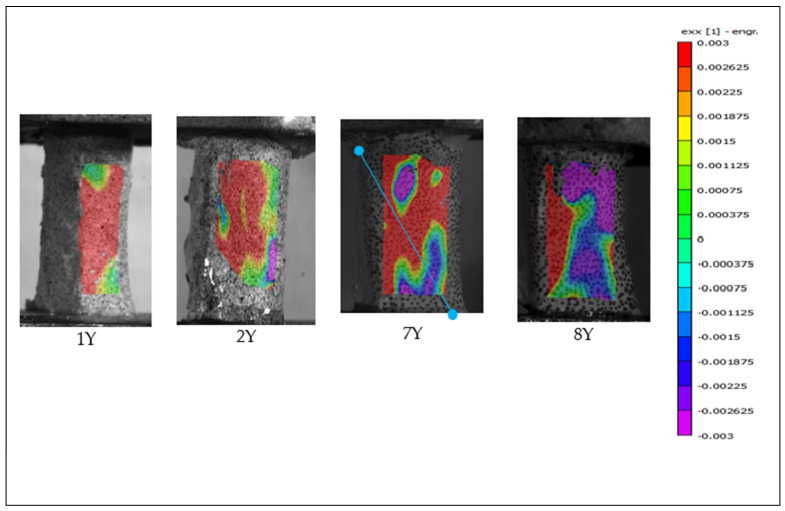
Full-field strain distribution in the X direction (exx) for the “Y” specimen obtained via digital image correlation, showing the frame immediately before fracture. The solid blue line indicates the spiral fracture path.

Of the 18 preliminary specimens that exhibited spiral fractures, digital image correlation (DIC) analysis was successfully performed on 12. The remaining six specimens could not be analyzed due to inadequate speckle pattern quality, which likely interfered with accurate strain tracking. Among all specimens, only specimen 7X failed within the camera’s field of view; the others fractured on the posterior side, outside the visible imaging area. Figure 5 and Figure 6 present the DIC results showing strain distribution in the X-direction (exx) for selected specimens from the primary test. Each frame captures the moment immediately preceding failure. The trajectory of the spiral fracture is indicated by a solid blue trace superimposed on the strain map.

### 3.4. Digital Image Correlation in Secondary Investigation

**Figure 7:**
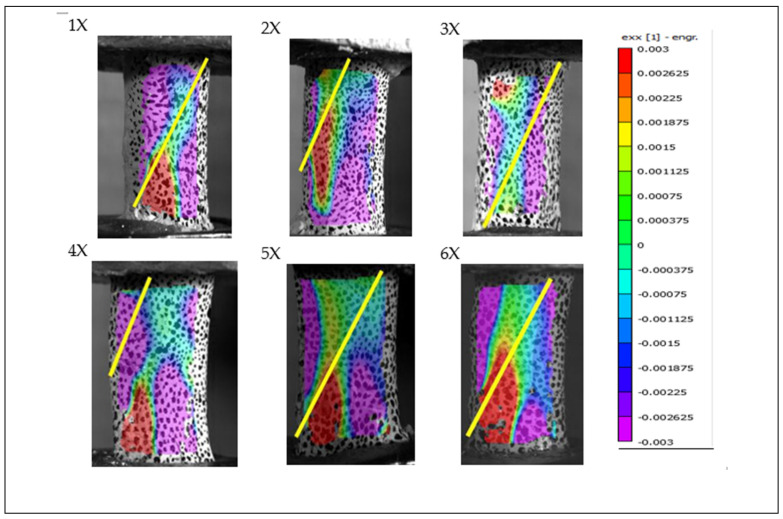
Full-field strain distribution in the X direction (exx) for the “X” specimen from the secondary test, obtained via digital image correlation. The image captures the frame immediately before fracture.

**Figure 8:**
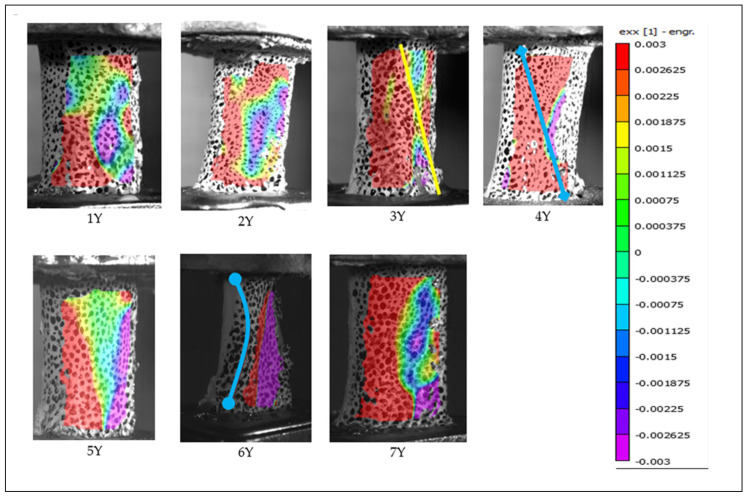
Full-field strain distribution in the X direction (exx) for the “Y” specimen from the secondary test, obtained via digital image correlation. The frame was captured immediately prior to fracture, with clear visualization of early failure in specimens 6Y and 7Y.

Digital image correlation (DIC) analysis was successfully performed on all specimens tested during the secondary investigation. All specimens classified as “X” (without periosteum) exhibited spiral fractures on the posterior surface of the bone, within the camera’s field of view. The fracture path for each specimen is indicated by a blue trace on the strain maps. Figure 7 and Figure 8 present the results of DIC analysis from the secondary test phase, highlighting strain distribution in the X-direction (exx) at the moment immediately preceding fracture. Notably, specimens 6Y and 7Y showed visible signs of structural compromise during the potting process, which are clearly illustrated in Figure 8.

### 3.5. Post Fracture Analysis

**Figure 9:**
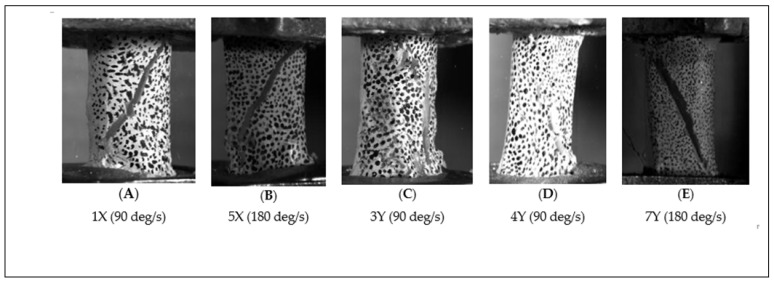
Post-fracture views of bone specimens subjected to torsional loading at 90°/s and 180°/s. Bones with periosteum are labelled “Y”, and those without periosteum are labelled “X”. (**A**–**D**) Specimens from the primary test; (**E**) Specimens from the secondary test.

Post-fracture analysis, as shown in Figure 9, compares fracture patterns in bones subjected to torsional loading at 90°/s and 180°/s. Specimens with intact periosteum are labelled “Y”, while those without periosteum are labelled “X”. Panels (A, B, C, D) and (E) represent the primary and secondary test phases, respectively.

**Figure 10:**
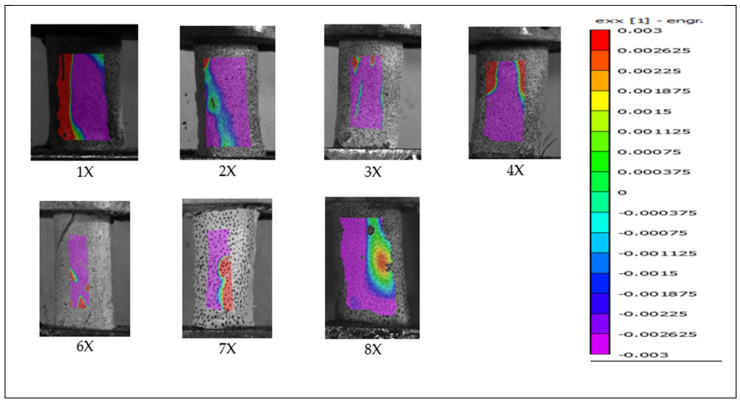
Compressive strain distribution observed post-failure in bones lacking periosteum (labelled “X”) from the primary test phase.

**Figure 11:**
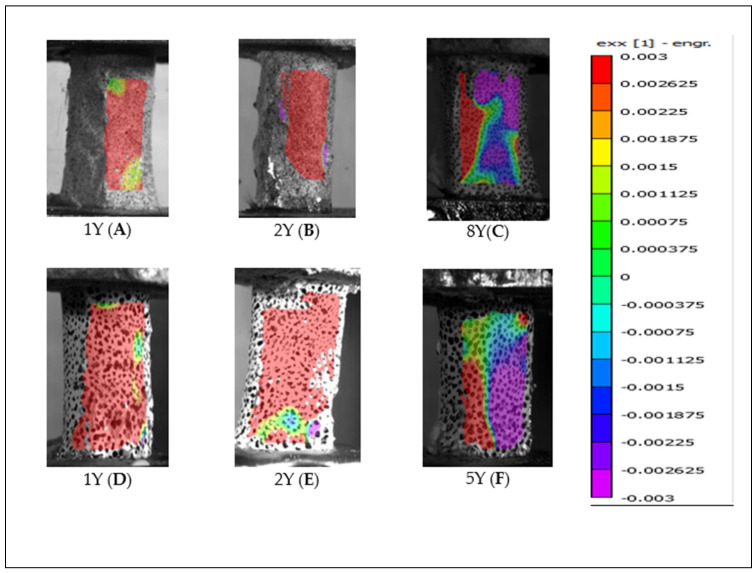
Tensile strain distribution observed post-failure in bones with periosteum (labelled “Y”) from both (**A**–**C**) the primary and (**D**–**F**) the secondary test phases.

Following failure, distinct differences in surface strain distribution were observed. As illustrated in Figure 10, bones lacking periosteum (“X”) exhibited increased compressive strain on the surface opposite the camera. In contrast, periosteum-intact specimens (“Y”) showed a pronounced increase in tensile strain on the same surface, as demonstrated in Figure 11. Panels (A, B, C) and (D, E, F) represent the primary and secondary test phases, respectively. This divergence in post-fracture strain behavior highlights the potential biomechanical role of the periosteum in modulating surface strain responses under torsional loading.

## 4. Discussion

In this study, more than 85% of the tested bones exhibited spiral fractures, including failures involving the growth plate or intercondylar region. High-speed digital image correlation (HS-DIC) analysis successfully confirmed spiral fracture patterns in the majority of these specimens. However, only about 45% of all samples fractured within the camera’s field of view, limiting the availability of full-field surface strain data for some cases.

Among the specimens with spiral fractures, approximately 85% were successfully analyzed using HS-DIC. Specimens 5X, 3Y, 4Y, 5Y, and 6Y from the primary test phase could not be analyzed, most likely due to suboptimal speckle pattern quality. As previously discussed, a high-quality speckle pattern should consist of a large number of randomly distributed, high-contrast, and high-density spots with an ideal diameter of approximately 2–4 pixels. In the initial tests, the airbrush technique occasionally produced fine droplets that resulted in low contrast against the white background, despite achieving high density. Additionally, achieving consistent spot size and distribution proved challenging.

In contrast, the hand-painted technique used in the secondary investigation provided better control over speckle size and positioning, resulting in improved contrast and reproducibility. This enhancement significantly improved the reliability of DIC analysis during the secondary testing phase.

The present findings on the biomechanics of long bone failure under torsion [20,28] align with the mean time to failure results shown in Figure 4 when comparing the two rotational speeds. Since stress accumulation within bone tissue is influenced by strain rate, and given that bones typically become stiffer and more resistant as strain rate increases, higher rotational speeds result in shorter times to failure. This behavior is consistent with the known viscoelastic properties of bone.

Digital image correlation (DIC) analysis of surface strain revealed greater regions of positive tensile stress on the fracture surface and higher negative compressive stress on the opposite side. These observations support the hypothesis that bone failure initiates in regions of high tensile strain, in line with the established understanding that bone tissue is strongest in compression and weaker in shear and tension [10].

Additionally, Figure 7 supports the observation that bone failure occurs primarily due to tensile strain rather than compressive forces. In all specimens shown in Figure 7, which depicts the frame immediately prior to failure in periosteum-removed bones from the secondary test, all fractures occurred within the camera’s field of view.

A distinct line of tensile strain (exx), extending from the bottom-left to the top-right corner of each specimen, is evident and surrounded by regions of compressive strain. The fracture consistently followed this diagonal tensile strain path, providing visual evidence of spiral fracture propagation along the axis of maximum tension.

Notably, the regions of highest tensile strain—marked in red—were located directly above the screw positioned in the lower pot, suggesting that localized stress concentration caused by the screw may have contributed to fracture initiation at that point. In contrast, such stress concentration was not observed in Figure 5, where no screw was placed through the bone in the lower fixture. However, it remains uncertain whether the absence of a stress concentration in Figure 5 is due to the screw’s absence or simply because the screw was located on the opposite, non-visible side of the bone.

Bones 5X and 6X in Figure 7, analyzed during the secondary test, provide additional visual evidence of the viscoelastic behavior of bone tissue. These two specimens, which were rotated at 180°/s, exhibited broader zones of neutral stress compared to those rotated at 90°/s. This observation may be attributed to the viscoelastic nature of bone, which tends to become stiffer and less responsive under higher strain rates, thereby resulting in wider neutral regions due to reduced deformation time.

However, this hypothesis requires further validation, as only two periosteum-free specimens fractured within the camera’s field of view at the higher rotation speed. Despite the limited sample size, these findings support the broader conclusion that bone failure is strain-rate dependent. Higher angular velocities are associated with faster and more brittle fracture behavior.

This strain-rate sensitivity underscores the clinical relevance of loading speed in real-world trauma scenarios, such as falls, vehicular collisions, and athletic injuries, where bones may experience rapid torsional forces that exceed their capacity to deform plastically.

The experimental protocol used in the secondary investigation, where cameras were focused on the anticipated fracture zone, proved more effective than the approach used in the primary test, particularly for bones without periosteum. However, predicting the precise failure location in periosteum-intact specimens remained challenging.

This difficulty may be attributed to the biomechanical role of the periosteum in distributing tensile stresses more broadly across the bone surface. As illustrated in Figure 6 and Figure 8 (vs. Figure 5 and Figure 7), periosteum-containing bones generally exhibited a wider dispersion of tensile strain before failure. This suggests that the periosteum may contribute to load redistribution, potentially delaying or diffusing fracture initiation. These observations underscore the importance of periosteal integrity in understanding fracture behavior and highlight the advantages of precise camera alignment in enhancing DIC data quality.

Figure 9 compares the fracture patterns of bones with and without periosteum at different rotational speeds, based on visual inspection independent of DIC analysis. For both 90°/s and 180°/s rotation rates, bones lacking periosteum (labeled “X”) exhibited rapid, brittle fractures characterized by a distinct diagonal fracture line. This pattern is consistent with torsional failure behavior in the absence of periosteal support.

Overall, bones without periosteum exhibited increased compressive stress on the surface opposite the fracture site, whereas bones with periosteum showed a post-failure increase in tensile stress. This contrast is evident in Figure 10 and Figure 11, which compare opposite sides of failed bones. In both periosteum-free specimens and those with periosteal disruption during high-speed rotation, continued twisting after the initial fracture appeared to cause additional deformation, driving one surface into compression and propagating the fracture in a spiral “peeling” pattern.

In contrast, periosteum-containing bones demonstrated periosteal bridging fibers spanning the fracture gap, placing the periosteum under increasing tension as the bone fractured, eventually leading to failure of the periosteal tissue itself, as observed in Figure 9. This behavior supports existing literature suggesting that the periosteum plays a biomechanical role in developing bone. Average torque values (Figure 3) consistently indicated that bones with periosteum failed at lower torques than those without, though these differences were statistically insignificant in most cases, except for one subset tested at 180°/s in the primary trial. These findings suggest that the periosteum may contribute more to post-failure stabilization, by maintaining alignment of bone fragments, than to pre-fracture mechanical strength.

One limitation of this study was the necessity of applying surface coatings for DIC analysis, which may have affected the native mechanical properties of the bone and periosteum. This is particularly relevant for periosteum-containing specimens, where the coating thickness may have approached or exceeded the periosteum’s own thickness. Future investigations should aim to replicate the experimental setup without coatings to assess their potential influence on fracture behavior and strain distribution.

While DIC could not be performed on all specimens, high-speed video recordings may still provide valuable qualitative insight into fracture dynamics and should be leveraged in future studies to validate observed failure modes.

Another limitation of this study was the frame rate used during high-speed imaging. Despite increasing the frame rate to 4000 frames per second, the complete fracture often occurred between two captured frames, making it impossible to visualize fracture propagation or pinpoint the exact initiation site. The digital image correlation (DIC) system was constrained to a maximum of 865 usable frames in sequence, which limited temporal resolution across the full fracture process.

To further assess the impact of surface coatings on mechanical behavior, particularly in immature bone specimens where the periosteum is thin, future studies should adopt both experimental and computational methodologies. Suggested directions include: (1) the quantitative assessment of coating thickness, using advanced imaging techniques such as optical profilometry and scanning electron microscopy (SEM); (2) the paired biomechanical testing of coated and uncoated bone specimens, comparing outcomes such as torque at failure, strain distribution, and fracture location; (3) high-speed imaging without surface coatings to directly visualize fracture initiation and propagation, and assess the effects of surface preparation; (4) Finite Element Modeling (FEM) to simulate stress propagation under different surface conditions and validate experimental findings [29,30]; (5) evaluation of alternative low-interference coatings, such as nanoparticle-based sprays, which preserve DIC contrast while minimizing structural impact; and (6) post-fracture histological and microscopic analysis to detect potential coating-induced alterations in bone or periosteal tissues.

Implementing these approaches may enhance the accuracy of biomechanical assessments and reduce artifacts associated with experimental preparation. Preliminary follow-up experiments are currently underway in our laboratory to investigate these aspects under controlled conditions, with the aim of validating the proposed methodologies and refining future experimental protocols.

## 5. Conclusions

This study provides new insights into the biomechanical role of the periosteum in immature long bones subjected to torsional loading. Using a paired experimental design and high-speed digital image correlation (HS-DIC), the investigation captured real-time surface strain distributions during spiral fracture formation. Clear differences in fracture behavior and strain localization were observed between bones with and without periosteum, particularly under varying rotation speeds.

Spiral fractures were reproduced in more than 85% of specimens. The presence of periosteum was associated with broader tensile stress zones and delayed fracture onset, suggesting a protective biomechanical function. These findings have important implications for pediatric trauma assessment, where periosteal integrity may modulate fracture patterns.

The study also confirmed that higher angular velocities result in significantly shorter times to failure, reflecting the viscoelastic nature of bone tissue. This underscores the importance of strain-rate sensitivity in both biomechanical experimentation and clinical trauma analysis.

Beyond its scientific findings, this work demonstrates the practical value of HS-DIC as a non-invasive, high-resolution technique for studying fracture mechanics in developing bone. Its ability to capture surface strain evolution with high temporal fidelity is particularly relevant for pediatric applications, where subtle structural differences can lead to markedly different failure behaviors.

By integrating HS-DIC with paired biomechanical testing, this study introduces a novel methodological framework with strong potential for clinical translation, particularly in trauma evaluation, orthopedic implant design, and forensic investigation. Future research combining biomechanical, histological, and material property analyses is encouraged to build upon these findings.

While no single fracture pattern can conclusively indicate abuse, the biomechanical characteristics identified in this study may aid in mechanism-based differentiation of pediatric fractures in both clinical and forensic settings.

## Figures and Tables

**Table 1:** Specimen coding and classification based on periosteum status and test phase.

Sample Code	Periosteum Status	Group
1X	With periosteum	Primary Test
2X	With periosteum	Primary Test
3X	With periosteum	Primary Test
4X	With periosteum	Primary Test
5X	With periosteum	Primary Test
6X	With periosteum	Primary Test
1Y	Without periosteum	Primary Test
2Y	Without periosteum	Primary Test
3Y	Without periosteum	Primary Test
4Y	Without periosteum	Primary Test
5Y	Without periosteum	Primary Test
6Y	Without periosteum	Primary Test
1X	With periosteum	Secondary Test
2X	With periosteum	Secondary Test
3X	With periosteum	Secondary Test
4X	With periosteum	Secondary Test
5X	With periosteum	Secondary Test
6X	With periosteum	Secondary Test
1Y	Without periosteum	Secondary Test
2Y	Without periosteum	Secondary Test
3Y	Without periosteum	Secondary Test
4Y	Without periosteum	Secondary Test
5Y	Without periosteum	Secondary Test
6Y	Without periosteum	Secondary Test

**Table 2:** Torsion test outcomes for the initial examination at 90°/s.

Specimen	Torque at Failure (N·mm)	Rotation Angle at Failure (°)	Time to Failure (s)
1X	21,083.87	22.08	0.3117
1Y	14,595.79	24.20	0.3326
2X	14,853.16	16.61	0.2741
2Y	17,079.79	17.75	0.2613

**Table 3:** Results of torsion testing for the main test at 180°/s.

Specimen	Torque at Failure (N·mm)	Rotation Angle at Failure (°)	Time to Failure (s)
3X	19,257.42	18.20	0.1692
3Y	18,181.79	17.43	0.1612
4X	17,094.86	20.44	0.1758
4Y	13,832.79	14.57	0.1438
5X	20,388.85	17.56	0.1708
5Y	16,013.89	12.83	0.1394
6X	20,773.25	20.63	0.1804
6Y	18,814.69	19.05	0.1753
7X	19,381.48	18.82	0.1738
7Y	15,534.71	17.91	0.1676
8X	19,389.41	19.27	0.1744
8Y	17,413.61	15.61	0.1532
9X	19,389.41	19.29	0.1750
9Y	17,413.61	15.70	0.1540

**Table 4:** Results of torsion testing for the secondary test at 90°/s.

Specimen	Torque at Failure (N·mm)	Rotation Angle at Failure (°)	Time to Failure (s)
1X	16,868.59	11.28	0.1913
1Y	12,974.59	−14.98	0.2293
2X	19,261.35	13.18	0.2072
2Y	-	-	-
3X	17,252.51	17.36	0.2515
3Y	16,932.79	−14.73	0.2253
4X	17,362.43	15.92	0.2360
4Y	15,812.11	−19.50	0.2789

Note: In the secondary test, rotation angles are presented as recorded by the testing machine. Counterclockwise rotations (bones with periosteum) are represented with negative values, and clockwise rotations (bones without periosteum) with positive values.

**Table 5:** Results of the torsion test for the secondary examination at 180°/s.

Specimen	Torque at Failure (N·mm)	Rotation Angle at Failure (°)	Time to Failure (s)
5X	13,659.12	14.40	0.1435
5Y	14,359.71	15.30	0.1472
6X	16,137.85	16.76	0.1568
6Y	-	-	-

## Data Availability

The data presented in this study are available on request from coauthor Dr. Ghaidaa A. Khalid. The data are not publicly available due to ethical restrictions.
